# 3-Amino-1,2,4-triazole Limits the Oxidative Damage in UVA-Irradiated Dysplastic Keratinocytes

**DOI:** 10.1155/2017/4872164

**Published:** 2017-12-13

**Authors:** Marina Tamara Nechifor, Diana Dinu

**Affiliations:** ^1^Department of Anatomy, Physiology and Biophysics, University of Bucharest, 91-95 Splaiul Independentei, 050095 Bucharest, Romania; ^2^Department of Biochemistry and Molecular Biology, University of Bucharest, 91-95 Splaiul Independentei, 050095 Bucharest, Romania

## Abstract

Reactive oxygen species (ROS) generated by UVA irradiation affect the keratinocyte cell membrane, DNA, and proteins and may cause serious injury to the skin. Treating human dysplastic keratinocytes (DOK) with 3-amino-1,2,4-triazole (AMT), a common catalase inhibitor, induced a compensatory mechanism for the hydrogen peroxide detoxification, which included a rise in glutathione peroxidase and glutathione reductase activities. Here, we examined a possible role of AMT in protecting a human DOK cell line against UVA-induced damage. In DOK cells exposed to UVA irradiation, we observed a substantial decrease in antioxidant enzymatic activities, such as catalase, glutathione peroxidase, glutathione reductase, and glutathione-S-transferase and an increase in lipid peroxidation and protein oxidation levels. Treating DOK cells with AMT prior to UVA exposure enhanced the activities of glutathione peroxidase, glutathione reductase, and glutathione-S-transferase, relative to nontreated cells. The enhanced antioxidant activities were correlated with decreased protein oxidation levels. Based on these results, we suggest that AMT may protect dysplastic keratinocytes against the harmful effects of UVA radiation.

## 1. Introduction

Exposure of human skin to solar light may lead to short- and long-term cutaneous photobiological effects, such as inflammatory skin disorders, premature skin aging, and skin cancer [1, 2]. Ultraviolet light A (UVA, 320–400 nm) accounts for approximately 90% of the sunlight UV waveband that reaches the Earth's surface; thus, its contribution to the harmful effects must be carefully considered. UVA radiation is penetrative and reaches targets well below the skin surface, acting on the basal layer of the epidermis, where proliferating keratinocytes reside [3]. Previous studies have demonstrated that UVA may cause substantial oxidative stress in skin cells [4, 5]. After its absorption by endogenous chromophores, UVA radiation may lead to the formation of reactive oxygen species (ROS), which in turn cause lipid peroxidation, protein oxidation, and DNA damage [6, 7]. There are many cellular chromophores acting as photosensitizers for UVA radiation, including flavins, melanin, free porphyrins, and heme-containing proteins such as cytochromes, peroxidases, and catalase [7]. When ROS formation exceeds a cell's biological defense capacity, oxidative stress occurs. Therefore, cellular antioxidant mechanisms in skin represent an important line of protection against UVA exposure. Among these defense mechanisms, skin cells rely on antioxidant enzymes, such as superoxide dismutase (SOD), catalase (CAT), glutathione peroxidase (GPX), peroxiredoxins (PRDX), and glutathione-S-transferase (GST) [8, 9]. Each of these enzymes has specific roles in preventing oxidative damage and all must be functional for optimal antioxidant protection, although some overlap exists. For instance, both CAT and GPX act on reducing hydrogen peroxide levels, and the exact contribution of CAT and GPX in decomposition of hydrogen peroxide produced in human cell remains subject to debate [10, 11]. One approach to elucidate the physiological function of each antioxidant enzyme during oxidative stress uses specific inhibitors. Specifically, 3-amino-1,2,4-triazole (AMT) is an inhibitor widely used for its controlled effects on catalase activity [12]. With this approach, several studies reported changes in oxidative stress markers and antioxidant defenses after AMT treatment in various animal models [13, 14] and in cell culture [15, 16]. Some of these studies reported compensatory responses to AMT-induced catalase depletion [17].

In the present study, we examined how AMT inhibition of CAT affected the activities of GPX, GR, and GST, and the oxidative damage of lipids and proteins in UVA-exposed dysplastic keratinocytes. Our results are first to show that AMT treatment of keratinocytes prior to UVA irradiation increased the activities of GPX, GR, and GST and limited the levels of protein carboxylation. Based on these results, we propose that AMT activates a compensatory enzymatic mechanism against UVA-induced oxidative stress in keratinocytes.

## 2. Materials and Methods

### 2.1. Cell Culture and Treatment

Human Caucasian dysplastic oral keratinocytes (DOK, ECACC number 94122104) were from Sigma-Aldrich (St. Louis, MO, USA). Cells were cultured at 37°C under a 5% CO_2_ humidified atmosphere in Dulbecco Modified Eagle's Medium (DMEM) supplemented with Ham F12 (3 : 1), 10% fetal bovine serum, 2 mM L-glutamine, and antibiotic antimycotic solution. Cells were seeded at 10^4^ cells/cm^2^ in 60 mm plastic Petri dishes and grown to 80% confluence. Prior to irradiation, the culture medium was removed. Cells were washed twice with 2 mL phosphate-buffered saline (PBS), left in 2 mL PBS, and irradiated from the top at a distance of 10 cm. For irradiation, we used a 365 nm UV lamp (model VL-340 BLB, Vilber Lourmat, France) at a light intensity of 381 *μ*W/cm^2^. Irradiation was performed for 1 h, which resulted in accumulated doses of 18.7 J/cm^2^, as measured [18] with a LaserStar Power Meter, provided with a 3A-P photodetector (Ophir Optronics Solutions Ltd., Jerusalem, Israel). To avoid thermal stimulation, UVA exposure was done in a ventilated laminar flow hood (Safeflow 1.8, Bioair, Siziano, Italy). Control cells were similarly handled but were shielded from UVA with an aluminum foil sheet. For preliminary investigation of the effect on catalase inhibition, cells were treated with several AMT concentrations, between 0.01 and 2 mM. To investigate the cumulative effect of AMT and UVA irradiation on markers of oxidative stress and the activities of antioxidant enzymes, cells were treated with 1.5 mM of AMT and then UVA irradiated for 1 h.

### 2.2. Cell Viability

Cell viability was evaluated using the neutral red technique [19]. From a 50 mg/l solution of neutral red (in culture medium), 3 mL was added to each dish. Cells were reincubated for 3 h at 37°C, resulting in the uptake of the vital dye into viable cells. The dye medium was removed and the cells were washed rapidly with 4% formaldehyde-1% CaCl_2_ to remove unincorporated dye. Neutral red was extracted into 3 mL of a 1% acetic acid-50% ethanol mixture. After 20 min, the absorbance was measured at 540 nm. The absorbance corresponding to the wells with control cells was set as the 100% viability value.

### 2.3. Intracellular Hydrogen Peroxide

Intracellular levels of hydrogen peroxide (H_2_O_2_) were analyzed using dihydrorhodamine 123 (DHR) as described by Huang et al. [20]. DHR, a nonfluorescent substance, can passively diffuse across membranes and in the presence of H_2_O_2_ is irreversibly oxidized to rhodamine 123, a green fluorescent compound. Confluent keratinocytes treated with DHR (10 *μ*g/mL) in DMEM for 30 minutes were collected by scraping and centrifugation. Cell pellets were resuspended in 1 mL PBS and then analyzed on a flow cytometer (Beckman Coulter, Inc., CA, USA) at excitation and emission wavelengths of 488 nm and 525 nm, respectively. The fluorescence signal from 10,000 cells was collected to calculate the mean fluorescence intensity of a single cell.

### 2.4. Protein Oxidation

Protein oxidation was evaluated by measuring carbonyl derivatives, the most common products of the covalent modification with the OxiSelect™ Protein Carbonyl ELISA Kit (San Diego, CA, USA). The protein carbonyls present in the sample or standard are derivatized to dinitrophenyl (DNP) hydrazone and probed with an anti-DNP antibody, followed by horseradish peroxidase (HRP) conjugated secondary antibody. The protein carbonyl content in the unknown sample is determined by comparing with a standard curve that is prepared from known reduced/oxidized bovine serum albumin (BSA) standards. The results were expressed as nanomoles of protein carbonyl per mg protein (nmole/mg).

### 2.5. Lipid Peroxidation

The level of lipid peroxidation was measured via the 2-thiobarbituric acid (TBA) color reaction for malondialdehyde (MDA), an end product of lipid peroxidation, by the modified method of Portolés et al. [21]. Cultured DOK cells were homogenized in 1 mL of 0.1 M saline phosphate buffer and sonicated on ice, at 40 V, 3 times for 30 seconds each. Then, 0.375 mL of 40% (w/v) trichloroacetic acid (TCA) and 0.2 mL of 0.1 M of TBA were added to lysates. The samples were incubated at 90°C for 30 min and a volume of 0.625 mL distilled water was added. Cells were centrifuged at 5000 rpm for 10 minutes. The level of lipid peroxidation in the supernatants was determined by the absorbance at 532 nm, using a MDA solution, freshly made by the hydrolysis of 1,1,3,3-tetramethoxypropane, as a standard. The results were expressed as nmoles of MDA per mL.

### 2.6. Enzymes Activity Assays

Harvested cells were homogenized with 50 mM potassium phosphate buffer (pH 7.5), 0.2% Triton X-100, and 0.5 mM phenylmethylsulfonyl fluoride (PMSF) and sonicated three times for 30 seconds on ice. Total cell lysates were centrifuged at 3000 rpm, 4°C for 15 minutes, and aliquots of the supernatant were used for subsequent enzymatic assays.

The CAT activity was assayed by monitoring the disappearance of H_2_O_2_ at 240 nm, according to the method of Aebi [22]. The CAT activity was calculated in terms of U/mg protein, where one unit (U) is the amount of enzyme that catalyzed the conversion of one *μ*mole H_2_O_2_ in a minute under standard condition of temperature, optimal pH, and optimal substrate concentration. Selenium-dependent GPX activity was measured by an indirect method [23], using tert-butyl hydroperoxide as substrate. This assay is based on the transformation of glutathione (GSH) to oxidized glutathione (GSSG) catalyzed by GPX, which is then coupled to the recycling of GSSG back to GSH utilizing glutathione reductase (GR) and reduced nicotinamide adenine dinucleotide phosphate (NADPH). The conversion of NADPH to NADP^+^ was followed by recording the changes in absorbance intensity at 340 nm, and the concentration of NADPH was calculated using a molar extinction coefficient of 6.22 × 10^3^ M^−1 ^cm^−1^. One unit of activity was defined as the amount of enzyme that catalyzes the conversation of one *μ*mole NADPH per minute, under standard condition.

The GR activity was measured according to the method of Goldberg and Spooner [24], in 0.1 M phosphate buffer, pH 7.4 with 0.66 mM GSSG, and 0.1 mM NADPH by recording the decrease of absorbance at 340 nm. The activity of this enzyme was expressed as mU/mg; one unit of GR activity has been calculated as one *μ*mole of NADPH consumed per minute under standard condition. The concentration of NADPH transformed under GR action was calculated using a molar extinction coefficient of 6.22 × 10^3^ M^−1 ^cm^−1^.

The GST (EC 2.5.1.18) activity was assayed spectrophotometrically, at 340 nm by measuring the rate of 1-chloro-2,4-dinitrobenzene (CDNB) conjugation with GSH, according to the method of Habig et al. [25], and calculated as mU/mg. One unit of GST activity was defined as the amount of enzyme that catalyzed the transformation of one *μ*mole of CDNB in conjugated product per minute. The extinction coefficient 9.6 mM^−1 ^cm^−1^ of was used for the calculation of CDNB concentration.

All enzymatic activities, calculated as specific activities (units/mg protein), were expressed as % relative to controls.

### 2.7. Protein Concentration

The protein concentration, expressed as mg/mL, was determined by the method of Bradford [26], using bovine serum albumin as a standard.

### 2.8. Western Blotting

Samples of 10 *μ*g protein were derivatized with 2,4-dinitrophenylhydrazine (DNPH) following the reagents and conditions described by Thiele et al. [27] and separated by 10% sodium dodecyl sulfate polyacrylamide gel electrophoresis (SDS-PAGE). Separated proteins were electrotransferred onto polyvinylidene difluoride (PVDF) membrane. The membranes were blocked in 5% blocking reagent in Tris buffered saline Tween (TBS-T) and incubated with a rabbit anti-dinitrophenylhydrazone antibody (Sigma-Aldrich, St. Louis, USA) in 1% TBS-T (1 : 150) for 18 h at 4°C. Primary antibody binding was detected by incubation with a peroxidase-conjugated secondary antibody (1 : 300) (Sigma-Aldrich, St. Louis, USA) for 1 h at room temperature. The membranes were then treated with the chemiluminescent substrate Luminata Forte (Merck Millipore, UK). The oxidized protein bands were detected with the Luminescent Image Analyzer (FujiFilm, UK) and quantified with GelQuant.NET software. Loading control was made using the same amount of samples separated by SDS-PAGE in the same conditions and stained with sensitive Coomassie Blue stain.

### 2.9. Statistical Analysis

Five replicates were performed to calculate the averages and standard deviations of the experimental data, while duplication was used to test for reproducibility of each applied assays.

Statistical analyses were carried out using analysis of variance (ANOVA). The post hoc comparisons between the means of the groups were done by Duncan's Multiple Range Test (DMRT). Data were analyzed using the SPSS^R^ for Windows computer program (Version 10.0). All data were expressed as means ± SD from analysis of duplicate of five independent experiments. The differences were considered significant at *p* < 0.05.

## 3. Results

### 3.1. Dose and Time Effect of AMT on Catalase

**Figure 1:**
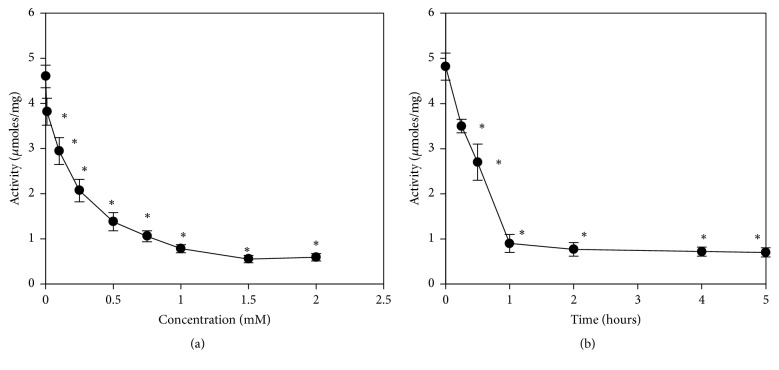
Dose- and time-dependent inhibition of CAT activity in keratinocytes treated with AMT. (a) Effect of several concentration of AMT; (b) effect of incubation time of DOK cells in culture media supplemented with 1.5 mM AMT. The values are calculated as means of five experiments performed in duplicate ± SD. *∗* indicates significant difference relative to controls (*p* < 0.05).

We incubated keratinocytes for 1 h with several AMT concentrations (0.01–2 mM) and measured a progressive decrease of CAT activity (Figure 1(a)). This effect was concentration-dependent with maximum inhibition observed at 1.5 mM AMT (Figure 1). The time course of CAT inactivation was observed during 24 h. The CAT activities had a deep decline up to an h of incubation. Thus, after 1 h of incubation with 1.5 mM AMT, the CAT activity was significantly reduced (approximatively 5-fold) compared to untreated cells (Figure 1(b)). This low level in CAT activity persisted up to 24 h of screening (Figure 1(b)). According to these results, the final chosen condition for the following experiments was 1.5 mM AMT for 1 h treatment.

### 3.2. Catalase Inhibition Induced Reactive Oxygen Species Generation in DOK Cells

Next we investigated the effect of AMT on ROS production and oxidative damage to lipids and proteins in DOK cells. As shown in Table 1, flow cytometric analysis showed that the mean fluorescence, that is, H_2_O_2_ production, was 2.2-fold increase in AMT-treated cells (Table 1). The treatment of DOK cells with 1 mM AMT resulted in an approximately 1.5-fold increase in lipid peroxidation content, while the amount of oxidatively damaged proteins showed only a minor increase (Table 1). The effect of AMT on the enzymes of the glutathione redox cycle was also investigated. The activity of GPX was upregulated by 150%, GR was increased by 68%, while GST activity remained almost unmodified after AMT treatment (Table 1).

### 3.3. Cell Viability

**Figure 2:**
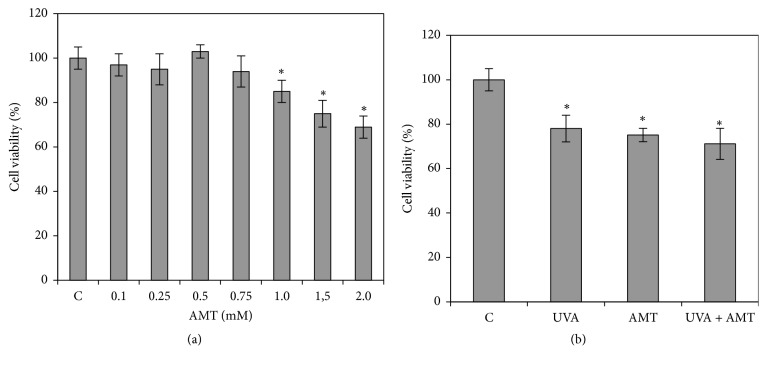
The viability of DOK cells exposure to AMT, at different concentrations (a), and the viability after UVA irradiation, AMT treatment, and the combined action of AMT treatment and UVA irradiation (b). The values are means ± SD from analysis of duplicate of five independent experiments and expressed as % from controls; ^*∗*^significantly different from controls (*p* < 0.05).

The incubation of DOK cells with 0.1, 0.25, 0.5, and 0.75 mM AMT for 1 h had no significant effect on viability. The decrease in cell viability induced by 1 mM AMT exposure was 14% from control. After the treatment of DOC cells with the highest of AMP concentrations of 1.5 and 2 mM, the viability decreased by 25% and by 31%, respectively, but statistical analysis showed no significant difference between them (Figure 2(a)).

To examine whether UVA, AMT, or AMT + UVA can lead to keratinocyte death, we performed cell viability assays. As shown in Figure 2(b), we noticed an approximate 25% decrease in cell viability after UVA irradiation and a similar decrease after AMT treatment, while in AMT-treated UVA-irradiated cells the loss in cell viability was about 29% from the control value.

### 3.4. The Antioxidant Enzymes

**Figure 3:**
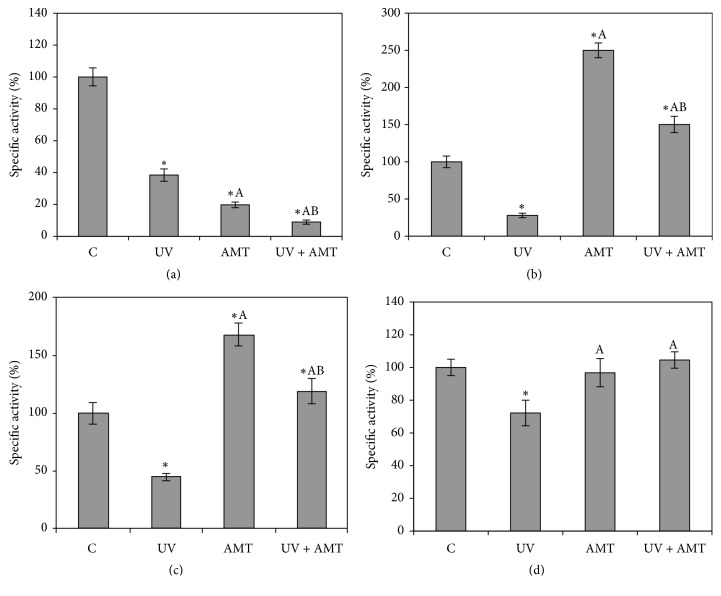
The effect of UVA exposure, AMT treatment, and concurrent AMT treatment and UVA exposure on antioxidant activities in DOK cells: CAT (a), GPX (b), GR (c), and GST (d). The values are calculated as means of five experiments performed in duplicate and expressed as % from controls; ^*∗*^significantly different from controls (*p* < 0.05); ^A^significantly different from UVA exposure (*p* < 0.05); ^B^significantly different from AMT treatment (*p* < 0.05).

Exposure of DOK cells to UVA, AMT, or AMT + UVA produced a significant decrease in CAT activities (Figure 3(a)). CAT activity decreased by 61.6% and by 80.3%, after UVA exposure and AMT treatment, respectively. The most dramatic loss in CAT activity was recorded in AMT-treated UVA-irradiated cells, where the residual CAT activity represented only 8.9% of control (Figure 3(a)).

For the other antioxidant enzymes, GPX and GR, we observed distinct responses relative to CAT (Figures 3(b) and 3(c)). Thus, we recorded a decrease in both enzyme activities after UVA irradiation, whereas AMT-treated cells had increased levels of these antioxidant activities. The incubation of DOK cells with AMT before UVA exposure not only prevented the depletion of GPX by UVA exposure but also enhanced the level of this enzyme by 50% compared to control cells. The treatment with AMT prior to irradiation also induced 18% increase in GR activity in UVA-irradiated cells. Regarding GST, the results revealed that the inhibition of GST by UVA irradiation was also prevented, if DOK cells were incubated with AMT prior UVA exposure, and the activity level remains similar to that recorded for the nonirradiated cells (Figure 3(d)).

### 3.5. Lipid Peroxidation and Protein Oxidation

**Figure 4:**
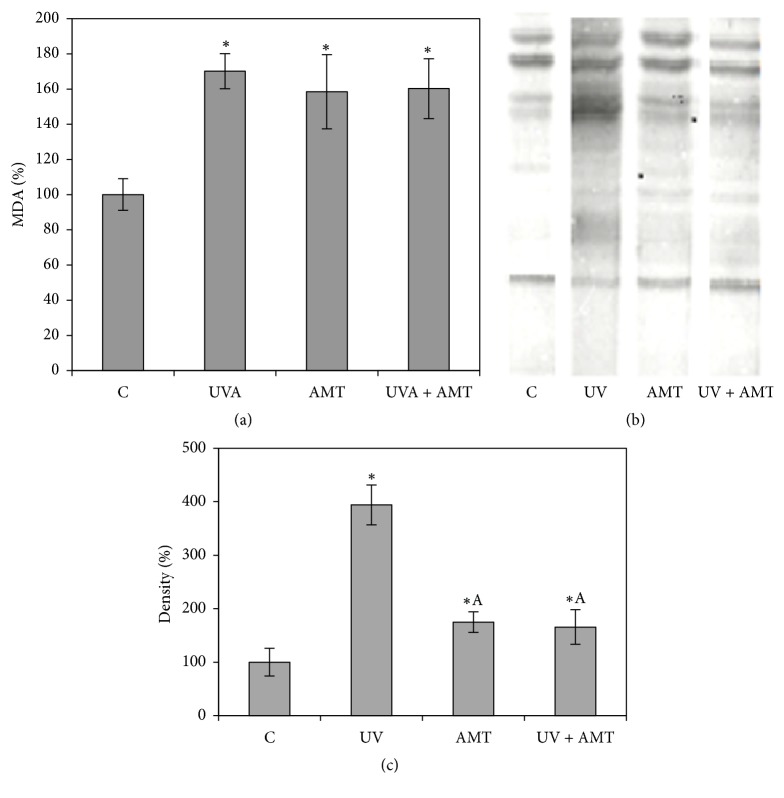
The effect of UVA exposure, AMT treatment, and concurrent AMT treatment and UVA exposure on lipid peroxidation (a) and on protein oxidation ((b), (c)) in DOK cells. (a) Values of MDA are means ± SD from analysis of duplicate of five independent cultures and are expressed as % from controls; (b) oxidized proteins detected by Western blot analysis: (c) the densitometry results for the level of oxidation of proteins were normalized to controls. The data are presented as means of three independent experiments ± SD; ^*∗*^significantly different from controls (*p* < 0.05); ^A^significantly different from UVA exposure (*p* < 0.05).

Lipid peroxidation levels, expressed as MDA, were significantly increased in UVA, AMT, and AMT + UVA treated cells, relative to control (Figure 4(a)). The most significant increase of 70% was recorded after UVA irradiation, but this high level was not significantly reduced through the treatment of keratinocytes with AMP prior to UVA exposure (Figure 4(a)). Following UVA exposure, a significant increase in protein oxidation occurred, as evident from the darker and/or new protein bands compared to nonirradiated cells, while protein carbonylation levels in AMT-treated cells were less affected (Figure 4(b)). The densitometry of the total oxidized proteins suggested a great increase in UVA-irradiated DOC cells, while the treatment of keratinocytes with AMT before irradiation significantly decreased the oxidative changes of proteins induced by UVA (Figure 4(c)).

## 4. Discussions

AMT is widely used to inhibit CAT activity and to investigate the physiological function of this antioxidant enzyme [12]. The removal of excess H_2_O_2_, which may result as a consequence of CAT inactivation, is very important to protect cellular components from oxidative damage.

At AMT concentrations between 0.1 and 1 mM a sharp reduction in CAT activity was seen in the preliminary data. CAT activity decreased by more than 80% in DOK cells after 1.5 mM AMT administration and remained at this level for higher concentrations of AMT. In addition, the decrease in DOK cells viability at this AMT concentration was moderate. Using these preliminary data, we chose treatment with AMT at a concentration of 1.5 mM as the design for the following experiments. In our experiments, we observed that depletion of CAT activity by AMT produced moderate oxidative stress in DOK cells. Specifically, the decrease of CAT activity by more 5-fold was accompanied by an increase of hydrogen peroxide level by only 2.2-fold. These data suggest the involvement of alternative mechanisms for detoxifying H_2_O_2_. Keratinocytes possess cellular defense systems, which, under normal metabolic conditions, regulate the level of ROS and protect against their deleterious effects. One of these defense systems includes antioxidant enzymes, such as CAT, GPX, GST, and GR [28]. CAT inactivation by AMT in keratinocytes was accompanied by the enhancement in GPX activity. The GPX enzyme works in tandem with CAT to remove hydrogen peroxide: CAT converts the hydrogen peroxide into molecular oxygen and water, while GPX uses reduced glutathione as an electron donor and catalyzes the biotransformation of various organic and inorganic peroxides [29]. We noticed that 150% increase in GPX activity could compensate, at least partially, the strong AMT-induced CAT inactivation, in terms of hydrogen peroxide removal. We also noticed an increase in GR activity in AMT-treated keratinocytes. The activation of GR, the enzyme, which catalyzes NADPH-dependent conversion of GSSG to GSH, has an important role in preventing the alteration of the glutathione status after AMT administration. The GST family contains enzymes that are capable of multiple reactions, with a multitude of substrates in order to detoxify endogenous compounds, such as peroxidized lipids; they are also involved in the metabolism of xenobiotics. Our results show that their activity remained unchanged after AMT treatment and therefore suggest that these enzymes are not involved in detoxification following AMT treatment. Previous reports indicated the activation of the enzymes involved in glutathione cycle after AMT treatment in animal model. Thus, a high level of GPX activity was noticed in goldfish brain, liver, and kidney after AMT administration [14, 17]. However, in our experiments, this protective response induced by CAT inactivation did not completely prevent the development of oxidative stress in keratinocytes. The level of lipid peroxidation increased by approximately 2-fold, while the protein oxidation rose by 1.3-fold. This difference between the magnitude of the changes registered for the two classes of macromolecules likely reflected their different susceptibilities to oxidative damage induced by ROS and possibly the intervention or different defense/reparatory mechanisms that counteract their oxidative changes in response to AMT treatment. Cells use different systems in order to provide antioxidant defense, damage removal, and replacement or repair for peroxidized lipids and oxidatively modified proteins. For example, glutathione peroxidase 4 acts as an efficient defense/reparatory enzyme reducing both soluble fatty acid hydroperoxides and also complex lipid hydroperoxides [30]. Peroxiredoxin 6 has also important roles in both antioxidant defense, based on its ability to reduce peroxidized membrane phospholipids, and reparatory mechanism (phospholipid homeostasis) based on its ability to generate lysophospholipid substrate for the remodeling pathway of phospholipid synthesis [31]. On the other hand, there are only few mechanisms involved in the recovery of the native state for oxidatively modified proteins: (i) intramolecular and intermolecular disulfide cross-links can be reversed to some extent by disulfide reductases; (ii) the enzyme methionine sulfoxide reductase can regenerate Met [32]. Protein carbonylation is considered an irreversible oxidative protein modification [33].

The compensatory response to CAT inhibition we observed in this study suggests that AMT might have potential value in modulating the effect of oxidative stress induced by various environmental factors, such as UVA radiation, when acting on skin cells. To understand the interference of AMT with the effects of UVA exposure in skin cells, we first attempted to evaluate the effect of AMT pretreatment on the viability of UVA-irradiated keratinocytes. Previous studies have shown that UVA exposure of cultured keratinocytes can lead to decrease in cell survival, depending on the irradiation intensity and exposure time [34, 35]. In the current work, we observed a moderate cytotoxicity after 18.7 J/cm^2^ of UVA exposure, measured as a decrease in cell viability of about 25%. In a study conducted by Huang et al. [20], the authors reported a decrease of HaCaT keratinocytes viability by 38% after 20 J/cm^2^ UVA exposure. AMT treatment also decreased cell viability, but the combined action of the two factors was not cumulative.

Using DOK cells as an* in vitro* model, our study showed that UVA irradiation decreased the level of the antioxidant defense enzymes. Therefore, the exposure of DOK cells to UVA radiation decreased significantly CAT, GPX, and GR activities, and, to a lesser extent, the GST level. In previous studies, such UVA-induced alterations in antioxidant enzymes have been also reported in cell culture experiments [36–38].

A very low level in CAT activity was recorded in AMT-treated and UVA-exposed DOK cells. Despite this effect, the harmful oxidative changes induced by UVA exposure were partially limited, probably due to the rise of GPX and GR activities, as a consequence of AMT treatment prior irradiation. The increase recorded in antioxidant enzymes activities was not enough to reduce the high level of UVA-induced lipid peroxidation. Thus, the AMT administration before irradiation, apparently cannot limit the undesirable consequences associated with UVA-induced lipid peroxidation.

UVA radiation is known to cause extensive protein modification [39]. Oxidation of protein side chains containing proline, arginine, lysine, and threonine results in the formation of carbonyl groups. In the current work, the quantification of protein profiles obtained suggested a sharp increase in carbonylated proteins after UVA exposure of keratinocytes. The increase in protein carbonylation upon UVA exposure was also reported in human dermis and epidermis [40] and in skin cell culture experiments [41]. Carbonylation of proteins is an irreversible oxidative damage, often leading to the accumulation of structurally and functionally impaired proteins [42]. Whereas moderately carbonylated proteins are degraded by the proteasome system, heavily carbonylated proteins form high-molecular-weight aggregates are resistant to proteasomal degradation and accumulate as damaged, unfolded proteins. Such aggregates seem to be involved in the pathogenesis of various skin diseases [43]. In our study, AMT treatment of DOK cells prior to UVA exposure decreased the UVA-induced protein oxidation. This observed decrease could have resulted from reduced ROS level, as a result of the AMT intensifying antioxidant enzymes activities.

In conclusion, AMT treatment of DOK cells induced a moderate oxidative stress as indicated by an increased level of MDA and carbonylated proteins. Although AMT reduced cellular CAT activity, the level of hydrogen peroxide and the extent of the oxidative damage were lower than expected, likely due to the compensatory activation of other antioxidant enzymes. Our results also showed that AMT treatment of DOK cells prior toUVA exposure limited the UVA-induced oxidative damage of proteins. A protective effect of AMT was previously reported. Thus, AMT effectively attenuated carbon tetrachloride-induced oxidative liver damage [44] and acetaminophen-induced mice hepatotoxicity were reported [45], but* in vitro* studies were not reported. Further mechanism-based studies are required to explain the interference of AMT with UVA irradiation in keratinocytes cells.

## Figures and Tables

**Table 1:** The effect of 1.5 mM AMT on intracellular H_2_O_2_ level, lipid peroxidation, protein oxidation, and antioxidant enzymes in DOK cells. ^*∗*^*p* < 0.05 versus control.

	Intracellular H_2_O_2_ level (main fluorescence)	Lipid peroxidation (nmoles/mL)	Protein oxidation (nmoles/mg)	GPX (U/mg)	GST (U/mg)	GR (U/mg)
Control	8.52	0.136	3.61	9.71	120.66	5.39
AMT	18.92^*∗*^	0.206^*∗*^	4.81^*∗*^	24.3^*∗*^	111.72	9.16^*∗*^
